# The Association Between Parents’ Knowledge About Human Papillomavirus and Their Intention to Vaccinate Their Daughters: A Cross-Sectional Study

**DOI:** 10.7759/cureus.48600

**Published:** 2023-11-10

**Authors:** Mona A Alshehri, Wafaa A Fahim, Rasha R Alsaigh

**Affiliations:** 1 Maternity and Childhood Nursing Department, King Abdulaziz University, Faculty of Nursing, Jeddah, SAU

**Keywords:** human papillomavirus, daughters, intention, parents, vaccine

## Abstract

Introduction

The human papillomavirus (HPV) causes the most sexually transmitted infections (STIs) worldwide. According to the World Health Organization (WHO), cervical cancer is the fourth most common type of cancer among women worldwide and the eighth leading cause of female cancer death in Saudi Arabia, especially in women between the ages of 15 and 44. The HPV vaccine is known to prevent HPV disease and death. Because parents are often the decision-makers regarding receiving HPV vaccination during adolescence, assessing parents’ knowledge about HPV and its relationship with their intention to vaccinate their daughters is highly necessary.

Materials and methods

An exploratory descriptive cross-sectional study design was used. A total of 773 parents, both mothers and fathers, of female students attending sixth grade from all areas of Jeddah city in Saudi Arabia were recruited. A self-administered validated questionnaire was used to collect the necessary data.

Results

The current study findings showed that 356 (46.1%) parents had poor knowledge about HPV, 119 (15.4%) had a fair level of knowledge, and 298 (38.5%) had a good level of knowledge. While 344 (44.5%) had intention to vaccinate, 337 (43.6%) were not sure and 92 (11.9%) were not intending to vaccinate. A significant association was identified between knowledge level and respondents’ variables such as being the father or mother, nationality, age, educational level, sector of employment, and monthly income. Employment status was significantly associated with the intention to vaccinate against HPV.

Conclusion

Concerns about the effectiveness and safety of the HPV vaccine as well as lack of knowledge about HPV and the vaccine influenced parents’ negative intention to obtain the vaccine. Health promotion initiatives for the HPV vaccine should be culturally responsive and emphasize the risks and benefits of the vaccine for women. Knowledge and attitudes about HPV can be improved through concise, visually designed, and comprehensive educational intervention programs targeting parents and their children at schools. Despite the proven safety and efficacy of HPV vaccines, more comprehensive strategies may be needed in the future to increase coverage rates of HPV vaccination nationwide.

## Introduction

The human papillomavirus (HPV) causes the most sexually transmitted infections (STIs) worldwide [1]. It has been linked to many cancers such as oropharyngeal, anal, vaginal, valvular, and especially cervical cancers [2]. The way of transmission of the virus is mainly by skin‐to‐skin or skin‐to‐mucosa via sexual transmission [3].

The most common high-risk HPVs are HPV-16 and HPV-18, which are associated with cervical cancer. HPV-6 and HPV-11 are the most common low-risk HPVs. Such infections can be threatening because many people who are infected do not manifest any symptoms, and the majority of HPV infections clear from the body without treatment [4].

According to the World Health Organization (WHO), cervical cancer is the fourth most common type of cancer among women worldwide, with an estimated 604,000 new cases of cervical cancer and 342,000 deaths reported in 2020. About 90% of new global infections and deaths in 2020 were in low- and middle-income countries [5].

People in Arab countries are suffering serious health problems because of increasing incidences of cervical cancer due to the limited availability of effective vaccination and screening programs for cervical cancer. One of the objectives of the strategy of the WHO to eradicate cervical cancer by the year 2030 is to vaccinate 90% of girls against HPV before turning 16 [6].

Also, in 2020, around 380 new cases of cervical cancer were diagnosed, and about 179 cervical cancer deaths occurred in Saudi Arabia. Cervical cancer is the eighth chief cause of female cancer death in Saudi Arabia and the eighth main cause of cancer death in women aged between 15 and 44 years old [7].

Vaccinations have played an important role in enhancing health and decreasing the global worry about vaccine-preventable diseases. Infection prevention is the utmost cost-effective public health approach for improving the health of the population. In 2016, the WHO identified the HPV vaccine as a public health priority that should be incorporated into national immunization programs [8]. The HPV vaccine offers multiple benefits, including preventing outbreaks and preventable diseases, reducing disease-related costs, and preventing the recurrence of infections that can cause cancer. Adolescents urgently need vaccination coverage to stop HPV disease and death due to HPV’s future complications [4]. Because parents are often the decision-makers regarding receiving HPV vaccination during adolescence, a detailed investigation of parental modifiable factors is needed.

Understanding the obstacles that parents who vaccinate their daughters face is essential if the HPV vaccine is to be used to its full potential to protect public health and prevent many HPV-related diseases and cancers. Information about vaccine safety and efficacy heavily influences parents’ choices. Parents who do not vaccinate their daughters often fear the vaccine’s side effects and doubt its efficacy, creating a barrier to vaccination [9].

Increased parental knowledge and awareness of HPV may increase motivation to have one’s daughters vaccinated against HPV, leading to improved vaccination coverage in Saudi Arabia [10]. Knowledge of HPV infection, the HPV vaccine, and HPV vaccination programs in the country plays a vital role in rising vaccination rates. A study conducted on the Saudi population showed reduced understanding and many misunderstandings regarding HPV and cervical cancer prevention [11]. The study revealed that only 10.9% of men and 15% of women had knowledge about HPV and cervical cancer. In another study conducted in Saudi Arabia, parents of about 70.6% of 296 participants did not know about the HPV vaccine, and most of them did not recognize the association of HPV with cervical cancer [12]. This study aimed to assess the association between parents’ level of knowledge about HPV and their intention to vaccinate their daughters, which can add to the dearth of literature in relation to Saudi Arabia.

## Materials and methods

Study design, setting, and sampling

An exploratory descriptive cross-sectional study design was used to assess the association between parents’ knowledge and their intention to vaccinate their daughters. After obtaining ethical approval from the Faculty of Nursing at King Abdulaziz University, official approval was obtained from the Department of Public Education in Jeddah city. A convenient sample of 773 parents was recruited from public primary schools in all areas including the north, south, east, and center of Jeddah city in Saudi Arabia between December 2022 and March 2023. The four main education offices in the city of Jeddah (North, Central, South, and East Jeddah) distributed the survey link to all parents registered in the system via email. Included parents, both mothers and fathers, had a daughter attending grade 6 at one of the primary government schools in Jeddah who were able to understand Arabic. While non-Arabic-speaking parents and those who did not complete the survey were excluded from the total sample. The self-administered survey was completed by parents after voluntarily consenting to participate, and they were fully aware of their right to confidentiality and withdraw at any time.

Data collection tools

A self-administered questionnaire was developed by the primary researcher after conducting an extensive review of the related literature. The distributed questionnaire had three parts.

Part I: Sociodemographic Characteristics of Parents

This part of the questionnaire covered parents’ demographic characteristics such as age, gender, nationality, marital status, number of children, educational level, field (related to the medical field or not) and occupation, monthly income, and family history of cervical cancer.

Part II: Parents’ Level of Knowledge

This part of the questionnaire assessed parents’ knowledge about HPV and the HPV vaccine. It measured parents’ level of knowledge using 21-item knowledge questions with “Yes,” “No,” and “Not sure” answer options. The scoring of the answers was as follows: “Yes” had a score of 1, and “No” or “Not sure” had a score of 0. The total scores ranged from 0 to 21. The scores were categorized as follows: 15-21, good knowledge; 8-14, fair knowledge; and 0-7, poor knowledge.

Part III: Parents’ Intention to Vaccinate Their Daughters

This part of the questionnaire identified parents’ intention to vaccinate based on 13 questions with “Yes,” “No,” and “Not sure” answer options. “Yes” had a score of 2, “Not sure” had a score of 1, and “No” had a score of 0. The score range was from 0 to 26. The scores were categorized as follows: 18-26, has the intention to vaccinate; 9-17, not sure; and 0-8, no intention.

The content validity of the questionnaire was assessed by three experts in the field of child and women’s health nursing who hold a doctoral degree. A pilot study was conducted with 77 (10%) parents. The Cronbach alpha coefficients of reliability were found to be 0.919 and 0.901 for knowledge and intention scales, respectively. The results confirm a high level of internal consistency of scale items. Therefore, no modifications were applied to the questionnaire.

Data analysis

The collected data were analyzed using Statistical Package for the Social Sciences (SPSS) version 25 (IBM SPSS Statistics, Armonk, NY). Descriptive statistics included frequency, percentage, and graphs used for categorical variables. The chi-squared test and Fisher’s exact test for association between categorical variables were conducted to examine the relation between HPV knowledge and intention to vaccinate based on the basic characteristics of participants. Spearman’s correlation was calculated between the scores for parents’ knowledge about HPV and their intention to vaccinate. Adjusted odds ratios (OR) of intention to vaccinate for HPV and 95% confidence interval (CI) of adjusted OR were calculated using a multinomial logistic regression model. A P-value of less than or equal to 0.05 was considered significant.

## Results

Demographic characteristics

As shown in Table 1, of the 773 parents, 604 (78.1%) were mothers. About half of them were Saudis (391 (50.6%)), and most of them were aged 36 years and above. The majority of parents (695 (89.9%)) were married, and 327 (42.3%) of them had at least a bachelor’s degree. Most of the parents were unemployed (490 (63.4%)), whereas 63 (14.6%) employed parents worked in the health sector. Nearly half of the parents (447 (57.8%)) had 4-7 children, and 487 (63%) of them reported that their monthly income was barely adequate. Most of the parents (745 (96.4%)) had no family history of cervical cancer.

**Table 1:** Sociodemographic characteristics

Variable	N = 773	%
Respondent		
Father	169	21.9
Mother	604	78.1
Nationality		
Saudi	391	50.6
Non-Saudi	382	49.4
Age of respondent		
25 or less	4	0.51
26-30	37	4.8
31-35	180	23.3
36 and above	552	74.1
Marital status		
Married	695	89.9
Divorced	61	7.9
Widow	17	2.2
Educational level		
Illiterate	18	2.3
Below secondary	109	14.1
Secondary	319	41.3
Bachelor’s degree and above	327	42.3
Employment status		
Employed	283	36.6
Not employed	490	63.4
If employed (n = 341)		
Health sector	63	14.6
Another sector	369	85.4
Number of children		
3 or fewer	285	36.9
4-7	447	57.8
8 or more	41	5.3
Monthly income		
Not enough	123	15.9
Barely enough	487	63
More than enough	163	21.1
History of cervical cancer		
Yes	28	3.6
No	745	96.4

Parents’ knowledge about HPV and the HPV vaccine

Table 2 clarifies that only 274 (35.4%) parents had heard of HPV infection, while only 136 (17.6%) of them asked for information about it, and 66 (8.5%) were given relevant information by the medical team.

**Table 2:** Parents’ knowledge about HPV and the HPV vaccine HPV: human papillomavirus

Item	Yes		No		Not sure	
	Number	%	Number	%	Number	%
Have you heard about HPV before this study?	274	35.4%	354	45.8%	145	18.8%
Have you ever asked for information about HPV?	136	17.6%	570	73.7%	67	8.7%
Have you been informed about HPV by the medical team?	66	8.5%	654	84.6%	53	6.9%
Is HPV a bacterial infection?	62	8%	155	20%	556	72%
Can HPV infection be treated with antibiotics?	67	8.7%	97	12.5%	609	78.8%
Can human papillomavirus infection cause cervical cancer?	201	26%	38	4.9%	534	69.1%
Does having sex at an early age increase the risk of infection with the human papillomavirus?	84	10.9%	74	9.6%	615	79.5%
Is HPV so rare?	82	10.6%	102	13.2%	589	76.2%
Does HPV always have obvious signs or symptoms?	78	10.1%	74	9.6%	621	80.3%
Are there many types of HPV?	88	11.4%	34	40.4%	651	84.2%
Is there currently a treatment or cure for HPV infection?	130	16.8%	41	5.3%	602	77.9%
Do you know how to get infected with human papillomavirus?	91	11.8%	300	38.8%	382	49.4%
Is the human papillomavirus transmitted through sexual contact only?	57	7.4%	110	14.2%	606	78.4%
Have you heard about the human papillomavirus vaccine?	246	31.8%	295	38.2%	232	30%
Have you ever asked for information about the HPV vaccine?	78	10.1%	535	69.2%	160	20.7%
Are HPV vaccines more effective if they are given to people who have not had sex before?	105	13.6%	57	7.4%	611	79%
Does the HPV vaccine protect your daughter from every type of HPV?	105	13.6%	62	8%	606	78.4%
Does the government provide the HPV vaccine?	237	30.7%	21	2.7%	515	66.6%
Are three doses required for the full HPV vaccine?	61	7.9%	37	4.8%	675	87.3%
Do you think there are any side effects of vaccination?	136	17.6%	45	5.8%	592	76.6%
Can HPV be prevented by vaccination alone?	58	7.5%	129	16.7%	586	75.8%

Based on the 21-item knowledge questions that measure parents’ level of knowledge about HPV and the HPV vaccine, Table 3 reports that 356 (46.1%) parents had poor knowledge about HPV and the HPV vaccine, 119 (15.4%) had fair knowledge, and 298 (38.5%) had good knowledge.

**Table 3:** Parents’ level of knowledge about HPV and the HPV vaccine HPV: human papillomavirus

Knowledge	Number	%
Poor	356	46.1
Fair	119	15.4
Good	298	38.5

Parents’ intention to vaccinate their daughters against HPV

Table 4 reports that 458 (59.3%) parents believe the vaccinations in general are a good idea and a way to protect public health, but only 286 (37%) were willing to give their daughter the HPV vaccine. However, more than half (418 (54.1%)) were not sure if the HPV vaccine has many benefits. In addition, 537 (69.4%) felt unsure about the vaccine having negative long-term effects on their daughters’ health. Furthermore, only 376 (48.6%) parents agreed that they needed to discuss the HPV vaccine with their daughters, while 269 (34.8%) were ready to vaccinate their daughters against HPV if the vaccination was freely available.

**Table 4:** Parents’ intention to vaccinate their daughters against HPV HPV: human papillomavirus

Item	Yes		No		Not sure	
	Number	%	Number	%	Number	%
I feel that vaccinations are a good way to protect public health.	458	59.3%	89	11.5%	226	29.2%
I feel that vaccinating children is a good idea.	424	54.9%	140	18.1%	209	27%
I am willing to give my daughter the HPV vaccine that can prevent HPV infection.	286	37%	235	30.4%	252	32.6%
I feel the HPV vaccine has many benefits.	247	31.9%	108	14%	418	54.1%
I feel the HPV vaccine will protect my daughter’s sexual health.	246	31.8%	105	13.6%	422	54.6%
I feel that the HPV vaccine is effective in preventing HPV.	246	31.8%	88	11.4%	439	56.8%
I feel that the HPV vaccine is effective in preventing HPV-related cancers.	224	29%	91	11.8%	458	59.2%
I feel that the HPV vaccine may lead to long-term health problems.	149	19.3%	87	11.3%	537	69.4%
I recommend the HPV vaccine to my daughter and relatives.	251	32.5%	171	22.1%	351	45.4%
I need to discuss the HPV vaccine with my daughter.	376	48.6%	139	18%	258	33.4%
I will vaccinate my children against HPV if the vaccination is freely available.	269	34.8%	194	25.1%	310	40.1%
I feel that information about HPV helps me decide whether to vaccinate my daughter against HPV.	414	53.6%	100	12.9%	259	33.5%
The HPV vaccine is so new that I want to wait a while before I make a decision if my daughter should get it.	402	52.1%	114	14.7%	257	33.2%

Based on the 13 questions in Table 4, parents’ intention to vaccinate was measured, as shown in Table 5. A total of 344 (44.5%) parents had the intention to vaccinate their daughters, 337 (43.6%) were not sure, and 92 (11.9%) were not intending to vaccinate at all.

**Table 5:** Parents’ intention to vaccinate their daughters against HPV HPV: human papillomavirus

Intention	Number	%
No intention	92	11.9
Not sure	337	43.6
Intention to vaccinate	344	44.5

HPV information sources

**Figure 1:**
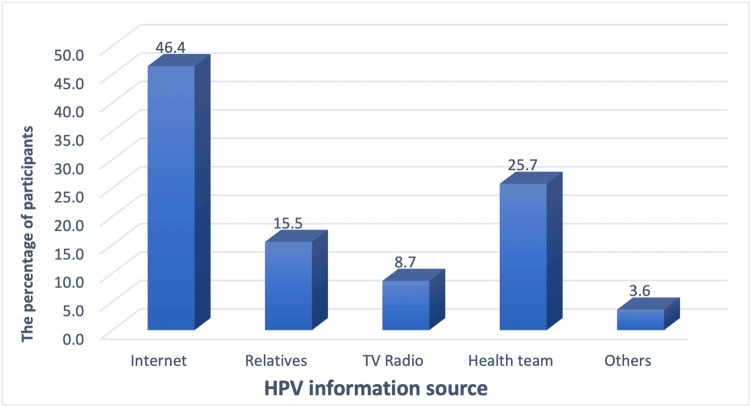
Distribution of HPV information sources HPV: human papillomavirus

Figure 1 shows the percentage distribution of HPV information sources. The internet was the main source of HPV information for 359 (46.4%) parents, followed by health teams (199 (25.7%)) and relatives and friends (120 (15.5%)).

Correlation between parents’ knowledge about HPV and the HPV vaccine and their intention to vaccinate

Table 6 shows the correlation between parental knowledge of HPV and the HPV vaccine and parents’ intention to vaccinate their daughters. The results indicated that the simple correlation coefficient between the two variables reached 0.209, which is a statistically significant value at the probability level of 0.01, indicating that there is a positive correlation between parents’ knowledge and parents’ intention to vaccinate their daughters.

**Table 6:** Correlation between parents’ knowledge about HPV and the HPV vaccine and their intention to vaccinate their daughters *Significant at 0.05 **Significant at 0.01 HPV: human papillomavirus

		Parents’ knowledge
Parents’ intention to vaccinate their daughters	Pearson’s correlation	0.209^**^
	Significance (two-tailed)	0.000
	Number	773

Basic characteristics of parents according to their level of knowledge about HPV and the HPV vaccine

Table 7 shows the results of using the chi-squared test for independence to examine the association between levels of knowledge and basic characteristics of parents. Significant associations were identified between knowledge level and variables such as parents being a father or a mother, nationality, age, educational level, sector of employment, and monthly income (P < 0.05).

**Table 7:** Basic characteristics of parents according to their level of knowledge about HPV and the HPV vaccine *Significant association ^1^Fisher’s exact test HPV: human papillomavirus

Variable	Poor		Fair		Good		χ^2^	P-value
	Number	%	Number	%	Number	%		
Respondent								
Father	63	37.3%	18	10.7%	88	52%	17.034	0.002*
Mother	293	48.5%	101	16.7%	210	34.8%		
Nationality								
Saudi	191	48.9%	73	18.7%	127	32.4%	14.419	0.007*
Non-Saudi	165	43.2%	46	12%	171	44.8%		
Age of respondent^1^								
25 or less	2	50%	2	50%	0	0%	12.414	0.041*
26-30	16	43.2%	6	16.2%	15	40.6%		
31-35	94	52.2%	32	17.8%	54	30%		
36 and above	244	44.2%	79	14.3%	229	41.5%		
Marital status								
Married	318	45.8%	110	15.8%	267	38.4%	1.770	0.778
Divorced	30	49.2%	8	13.1%	23	37.7%		
Widow	8	47.1%	1	5.8%	8	47.1%		
Educational level								
Illiterate	6	33.3%	3	16.7%	9	50%	21.812	0.001*
Below secondary	55	50.5%	8	7.3%	46	42.2%		
Secondary	137	42.9%	41	12.9%	141	44.2%		
Bachelor’s degree and above	158	48.3%	67	20.5%	102	31.2%		
Employment status								
Employed	124	43.8%	46	16.3%	113	39.9%	0.920	0.631
Not employed	232	47.4%	73	14.9%	185	37.8%		
If employed (n = 341)								
Health sector	33	52.4%	16	25.4%	14	22.2%	8.502	0.014*
Another sector	162	43.9%	58	15.7%	149	40.4%		
Number of children								
3 or fewer	137	48.1%	51	17.9%	97	30%	8.918	0.063
4-7	205	45.9%	58	13%	184	41.1%		
8 or more	14	34.2%	10	24.4%	17	41.4%		
Monthly income								
Not enough	49	39.8%	16	13%	58	47.2%	12.469	0.014*
Barely enough	235	48.3%	66	13.5%	186	38.2%		
More than enough	72	44.2%	37	22.7%	54	33.1%		
History of cervical cancer								
Yes	14	50%	3	10.7%	11	39.3%	0.516	0.772
No	342	45.9%	116	15.6%	287	38.5%		

Basic characteristics of parents according to their intention to vaccinate against HPV

Table 8 shows the results of using the chi-squared test for independence to examine the association between parental intention to vaccinate against HPV and the basic characteristics of parents. Employment status was significantly associated with intention to vaccinate against HPV (P < 0.05).

**Table 8:** Basic characteristics of parents according to their intention to vaccinate against HPV *Significant association ^1^Fisher’s exact test HPV: human papillomavirus

Variable	No		Not sure		Intended		χ^2^	P-value
	Number	%	Number	%	Number	%		
Respondent								
Father	12	7.1%	74	43.8%	83	49.1%	5.224	0.073
Mother	80	13.3%	263	43.5%	261	43.2%		
Nationality								
Saudi	43	11%	166	42.5%	182	46.5%	1.524	0.466
Non-Saudi	49	12.8%	171	44.8%	162	42.4%		
Age of respondent^1^								
25 or less	0	0%	1	25%	3	75%	8.057	0.221
26-30	6	16.2%	17	46%	14	37.8%		
31-35	24	13.3%	65	36.1%	91	950.6%		
36 and above	62	11.2%	254	46%	236	42.8%		
Marital status								
Married	85	12.2%	300	43.2%	310	44.6%	3.578	0.4661
Divorced	6	9.8%	26	42.6%	29	47.6%		
Widow	1	5.9%	11	64.7%	5	29.4%		
Educational level								
Illiterate	4	22.2%	8	44.5%	6	33.3%	6.828	0.337
Below secondary	11	10.1%	47	43.1%	51	46.8%		
Secondary	37	11.6%	152	47.7%	130	40.7%		
Bachelor’s degree and above	40	12.2%	130	39.8%	157	48%		
Employment status								
Employed	23	8.1%	123	43.5%	137	48.4%	6.878	0.032*
Not employed	69	14.1%	214	43.7%	207	42.2%		
If employed (n = 341)								
Health sector	4	6.4%	23	36.5%	36	57.1%	3.063	0.216
Another sector	39	10.6%	161	43.6%	169	45.8%		
Number of children								
3 or fewer	33	11.6%	122	42.8%	130	45.6%	0.361	0.985
4-7	54	12.1%	198	44.3%	195	43.6%		
8 and above	5	12.2%	17	41.5%	19	46.3%		
Monthly income								
Not enough	12	9.8%	66	53.7%	45	36.5%	6.930	0.139
Barely enough	57	11.7%	207	42.5%	223	45.8%		
More than enough	23	14.1%	64	39.3%	76	46.6%		
History of cervical cancer								
Yes	2	7.1%	10	35.7%	16	57.2%	2.010	0.366
No	90	12.1%	327	43.9%	328	44%		

Multinomial logistic regression model of intention to vaccinate (reference: no intention)

Table 9 presents the results of the multinomial logistic regression model. The intention to vaccinate is the dependent variable with three categories: no intention, not sure, and intention to vaccinate. The “no intention” category was set as a reference category.

**Table 9:** Multinomial logistic regression model of intention to vaccinate (reference: no intention) Note: “No intention” is the reference category. Chi-squared probability = 0.001, pseudo-R-squared = 0.317 OR: odds ratio, CI: confidence interval

Category	Variable	Adjusted OR	P-value	95% CI
Not sure	Knowledge			
	Poor (reference)	-	-	-
	Fair	0.98	0.974	0.45-2.14
	Good	2.72	0.049	0.35-4.45
	Respondent			
	Father (reference)	-	-	-
	Mother	0.76	0.562	0.31-1.89
	Marital status			
	Married	0.94	0.660	0.65-1.89
	Divorced	0.89	0.637	0.72-2.23
	Widow (reference)	-	-	-
	Educational level			
	Illiterate (reference)	-	-	-
	Less than secondary	0.45	0.410	0.21-1.65
	Secondary	0.85	0.328	0.65-1.86
	Bachelor’s degree and above	1.12	0.769	0.61-2.45
	Employment status			
	Employed (reference)	-	-	-
	Not employed	0.52	0.129	0.22-1.21
	If employed			
	Health sector (reference)	-	-	-
	Another sector	0.68	0.526	0.21-2.17
	History of cervical cancer			
	Yes (reference)	-	-	-
	No	2.56	0.197	0.52-3.36
Intention to vaccinate	Knowledge			
	Poor (reference)	-	-	-
	Fair	1.11	0.124	0.51-2.41
	Good	1.24	0.045	1.11-2.87
	Respondent			
	Father (reference)	-	-	-
	Mother	0.57	0.227	0.23-1.41
	Marital status			
	Married	1.51	0.032	1.11-3.32
	Divorced	1.41	0.025	1.13-2.87
	Widow (reference)	-	-	-
	Educational level			
	Illiterate (reference)	-	-	-
	Less than secondary	0.62	0.609	0.24-2.21
	Secondary	1.18	0.322	0.87-2.45
	Bachelor’s degree and above	1.12	0.627	0.56-3.21
	Employment status			
	Employed (reference)	-	-	-
	Not employed	0.62	0.262	0.27-1.42
	If employed			
	Health sector (reference)	-	-	-
	Another sector	0.46	0.176	0.15-1.41
	History of cervical cancer			
	Yes (reference)	-	-	-
	No	0.84	0.381	0.16-3.21

The overall model is found to be highly significant, as indicated by the P-value of the chi-squared statistic for the difference in the -2-log likelihood between the final model and the reduced model. The pseudo-R-squared value suggests that 31.7% of the variation in the dependent variables is explained by the variance in the explanatory variables included in the model.

The results of the “not sure” category reported that knowledge is a significant predictor of being not sure about vaccination. Participants with good knowledge were 2.72 times more likely to be not sure of having vaccination compared to others.

Regarding the predictors of intention to vaccinate, the variables knowledge and marital status were found significant. Participants with good knowledge were 1.24 times more likely to have vaccination.

## Discussion

HPV is a prevalent STI that has been linked to a variety of cancers. Vaccination is a safe and effective method of preventing HPV [13]. The Saudi Ministry of Health has recently started to launch a campaign targeting middle school female students to vaccinate them against HPV. According to the researcher’s knowledge, this study is one of the first studies in Saudi Arabia to examine the relationship between parents’ knowledge about HPV and their intention to vaccinate their daughters. In Saudi Arabia, parents make the decision about whether to vaccinate their children against HPV. The decision is complex, and several factors must be considered. Sociodemographic factors, attitudes, beliefs, knowledge, and religious and cultural factors influence individual decisions [14,15]. Government policies and access to proper health services (e.g., health examinations, vaccinations, and screening programs) are important considerations at the national level [16].

Parents’ knowledge about the benefits of the HPV vaccination was generally poor. Nearly half of the Saudi parents had low knowledge of HPV and its vaccine, which is in line with the results of other studies conducted in Saudi Arabia [11,17,18]. The lack of knowledge among Saudi parents about HPV and its vaccine may be due to a lack of extensive educational campaigns, especially in primary healthcare centers and public schools, and parents’ reliance on the basic vaccinations listed in their children’s vaccination cards, which does not include the HPV vaccines. Nevertheless, it is good that despite having low knowledge about HPV, Saudi parents support vaccination against HPV and other STIs. The lack of knowledge about HPV infection and its vaccine has been previously reported across Arab countries such as Bahrain, Saudi Arabia, the United Arab Emirates, Egypt, Morocco, Sudan, Lebanon, Jordan, and Syria [19]. Also, a lack of willingness to vaccinate was also reported in these countries [9]. Concerns about insufficient knowledge about the HPV vaccine have been linked to unwillingness to take the vaccine in this region [20,21]. Also, fear of side effects was found to be one of the most significant factors in the unwillingness to take the HPV vaccine [22,23]. This might be due to the lack of knowledge about HPV and its vaccine, especially around the safety of the vaccine. Therefore, great disparities in knowledge and willingness to vaccinate across countries in the Middle East might still be an ongoing issue that needs to be addressed promptly.

In addition to nationality, other correlations were identified between significant knowledge of HPV and respondent variables such as being a father or a mother, age, educational level, work sector, and monthly income. The current study noted that fathers had a slightly higher knowledge and intention to vaccinate their daughters compared to mothers. However, the number of fathers who participated was much lower than the number of mothers. This contradicts the results of another study, in which mothers were 1.4 times more likely than fathers to agree to obtain the HPV vaccine [24]. However, it is important to note that the acceptance rate of HPV vaccination can vary greatly depending on individual knowledge and attitudes toward the vaccine [25]. Therefore, it is crucial to provide accurate and comprehensive information about HPV and the HPV vaccine to both parents to ensure informed decision-making. Although separately exploring the association between the basic characteristics of mothers and fathers according to their knowledge level and intention to vaccinate their daughters against HPV was out of the scope of this paper, the researcher highlights the need for such exploration.

Monthly income was associated with acceptance of the HPV vaccine. Fathers with high incomes were more accepting of the vaccine, consistent with other study findings [26,27]. Meanwhile, parents with lower monthly incomes were less eager to vaccinate their daughters. This could be attributed to their lack of exposure to mass media messaging, health literacy, and the effects of globalization and urbanization as health services and education can be obtained easier and faster in cities than in rural areas. This study urges initiatives to consider the low socioeconomic population’s knowledge gaps about cervical cancer and HPV vaccination.

Surprisingly, in this study, the results of knowledge about HPV of parents who were employees in the non-health sector was higher than those working in the health sectors. However, parents who were health workers were more willing to vaccinate their daughters. This is also reflected in a study conducted to examine knowledge about cervical cancer among health practitioners in King Fahad Medical City, which showed that awareness about cervical cancer among the practitioners was poor. The authors highlight the importance of establishing awareness programs to educate healthcare workers about cervical cancer and HPV [28]. Meanwhile, a study in Bahrain indicated that employment in the health sector is a determining factor in HPV awareness [22].

The internet was found in this study to be the most common source where parents sought information about HPV. Social media messages are considered a reliable source of information in Saudi Arabia [17,28]. However, there is a possibility of misinformation being spread on social media. As a result, this study highlights the need for coordinated efforts to develop appropriate media content about HPV and the HPV vaccine to bridge the gap in the general Saudi population’s knowledge.

The current study found that most participants were not confident about the safety of the vaccine. Several other studies found that the main reasons for mothers’ refusal of the HPV vaccine were concerns about its safety and its negative effects on young adolescents [29]. It is the responsibility of healthcare providers to educate parents and emphasize the safety of HPV vaccines by providing easy-to-understand scientific evidence.

The study also found another major reason for parents’ reluctance to vaccinate their daughters against the HPV vaccine, which is the lack of recommendations from healthcare providers. It was noted that the majority of the parents in this study did not receive recommendations from their child’s healthcare provider to get the vaccine, which is in line with the findings of the study of Cunningham-Erves et al. (2018) [30]. This raises the necessary need for healthcare providers to raise awareness among their clients at the individual and community level to reduce the burden of HPV infection and reduce HPV-related cancers.

The predictors of intention to vaccinate as discussed previously can assist policymakers, the Ministry of Health, and vaccination campaign leaders in planning a delivery strategy to maximize reaching the entire segment of the population putting in mind the factors that account for parents to have no intention or hesitancy to vaccinate their daughters, such as lack of knowledge and awareness about the vaccine and its side effects, employment, and marital status.

Demographic variables and individual differences explained the percentage of variance in vaccination intention in the parents of this study. These interesting findings should be investigated further to look at other factors that account for non-intention to vaccinate such as cultural beliefs.

Healthcare workers at primary healthcare clinics and school health visits should emphasize the need and safety of the vaccine targeting parents and their daughters to help increase HPV vaccination uptake in the country. Future research should include investigations into the actual uptake and completion rates of HPV vaccination and the barriers to obtaining the HPV vaccine in different regions of Saudi Arabia.

Healthcare providers must be taught about HPV, its symptoms, and treatment, as well as the ways to prevent it, with a focus on the HPV vaccine. The role of healthcare centers in raising awareness and disseminating accurate and correct information about HPV and its vaccination should be emphasized. Health promotion programs for the HPV vaccine and warnings about the risks of HPV infection in women should be implemented. The benefits of the HPV vaccine should be taught in all government and private schools across the country, keeping cultural sensitivity in mind.

Although this study is one of the first studies in Saudi Arabia to assess parental knowledge and intention to vaccinate against HPV using a large number of participants recruited from government schools covering the perspective of both parents, mothers and fathers, the limitation of this study is that the sample consisted solely of parents from only Jeddah city in Saudi Arabia. This may limit the generalizability of the findings to other parts of the country. Future research could address these limitations by exploring larger and more diverse samples from across Saudi Arabia.

## Conclusions

Concerns about the effectiveness and safety of the vaccination as well as a lack of knowledge about HPV and the HPV vaccine influenced parents’ negative intention to obtain the HPV vaccine. Health promotion initiatives for the HPV vaccine should be culturally responsive and should emphasize women’s risks of experiencing an HPV infection as well as the benefits of getting vaccinated. Knowledge and attitudes about HPV can be improved through a concise, visually designed, and comprehensive educational intervention experience targeting children in schools and their parents. Despite the proven safety and efficacy of HPV vaccines, more comprehensive strategies may be needed in the future to increase coverage rates of HPV vaccination nationwide.
